# Supplementary material for: A chemical screen for modulators of mRNA translation identifies a distinct mechanism of toxicity for sphingosine kinase inhibitors
Source: PLoS Biol. 2021 May 25;19(5):e3001263. doi: 10.1371/journal.pbio.3001263 (PMC8183993; doi:10.1371/journal.pbio.3001263)
Supplement: S1 Raw images — (PDF) [file pbio.3001263.s016.pdf]

Figure 2

2C

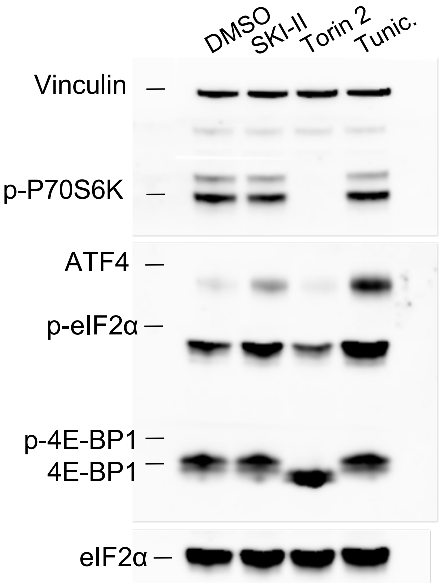

2E

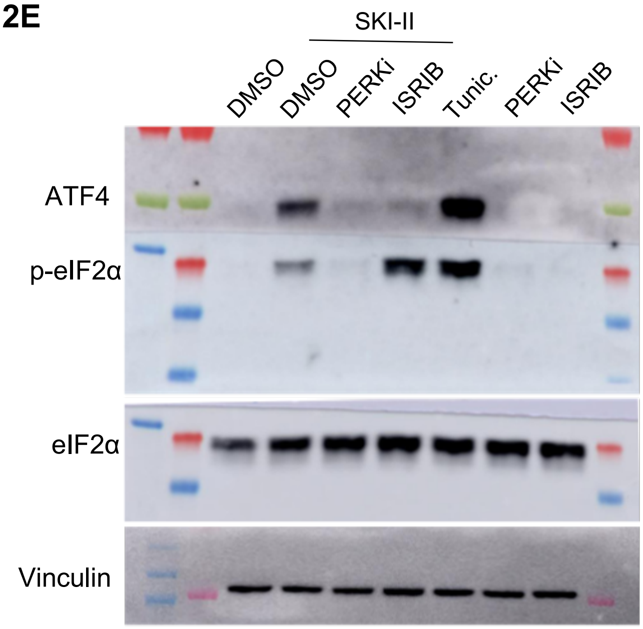

(Below) The samples were additionally run in a 3-8% Tris-Acetate gel for better resolution of PERK shift

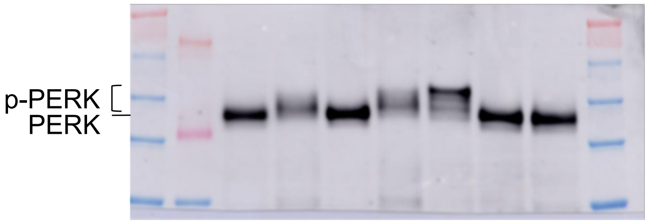

3-8% Tris-Acetate Gel

Figure 4

4E

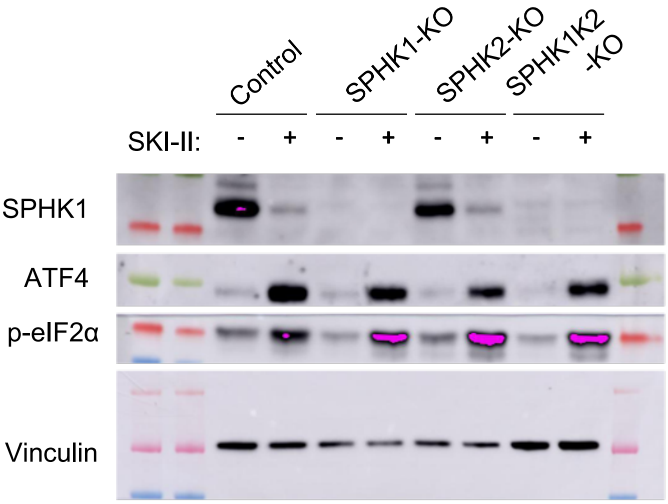

Jes-Wes system

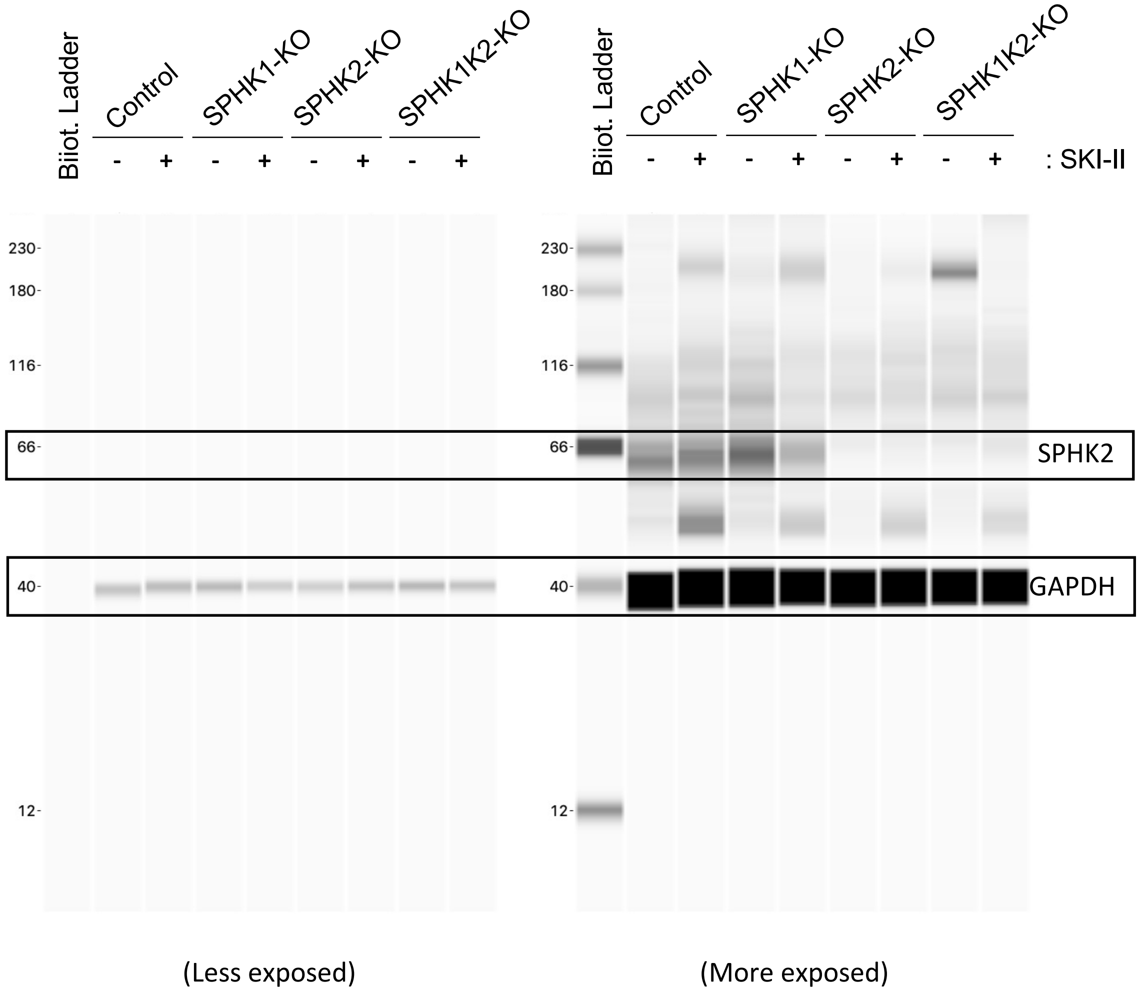

Supp. Figure 1

1C

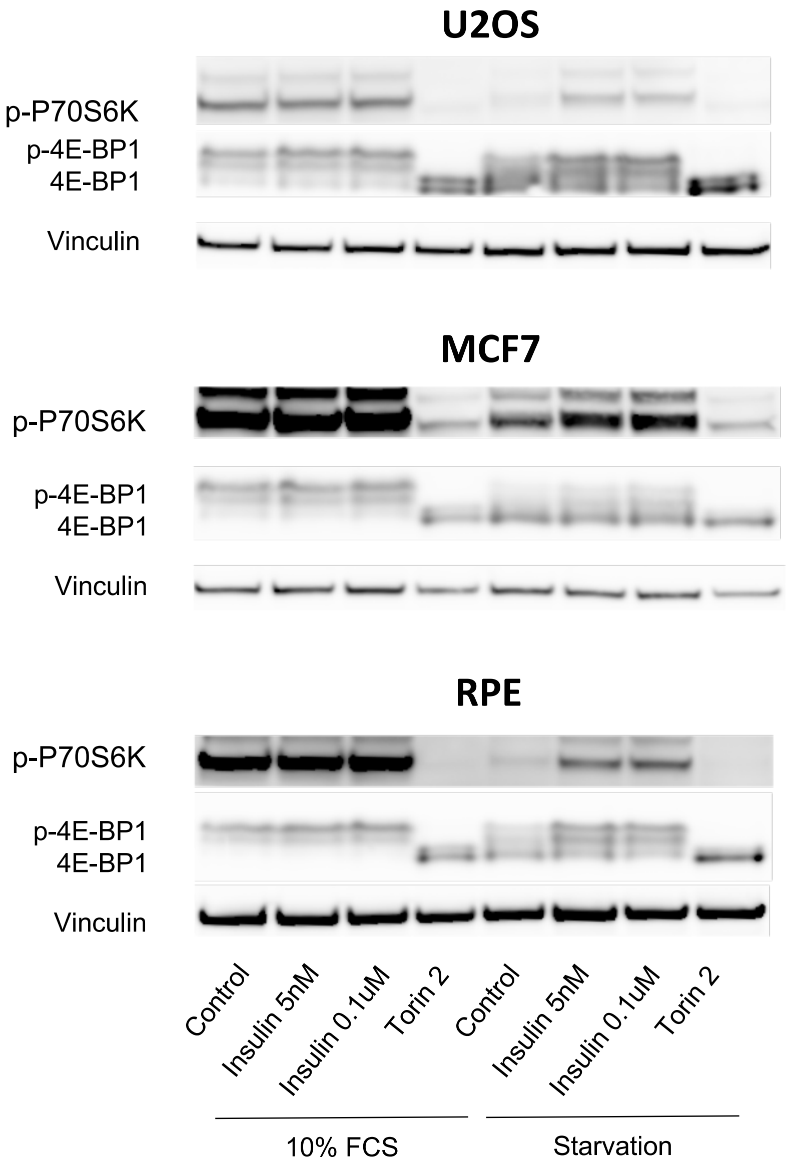

Supp. Figure 4

4C

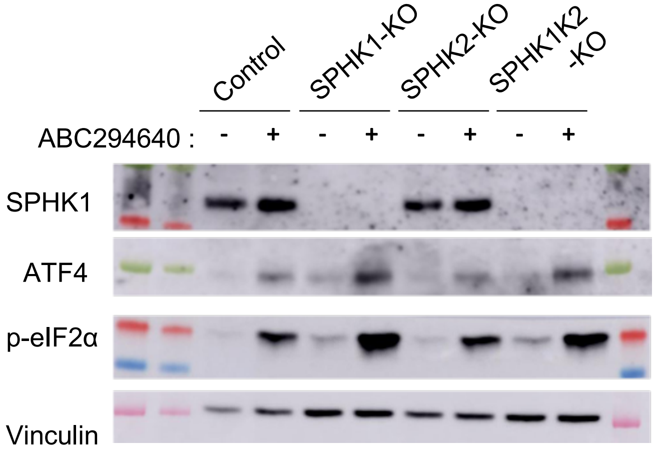

Jes-Wes system

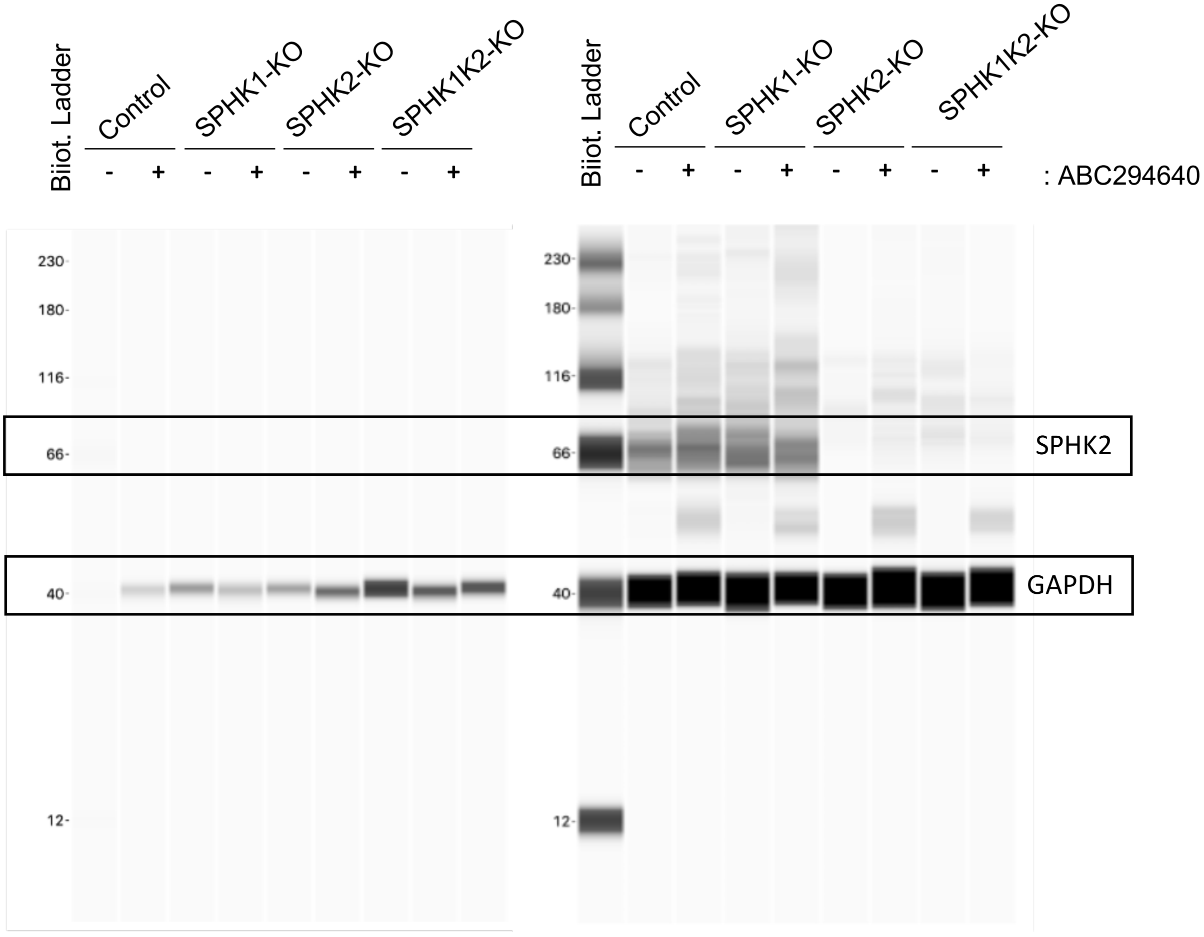

(Less exposed)

(More exposed)
