# Supplementary material for: A chemical screen for modulators of mRNA translation identifies a distinct mechanism of toxicity for sphingosine kinase inhibitors
Source: PLoS Biol. 2021 May 25;19(5):e3001263. doi: 10.1371/journal.pbio.3001263 (PMC8183993; doi:10.1371/journal.pbio.3001263)
Supplement: Attachment — Submitted filename: Point_by_point[OPP].pdf [file pbio.3001263.s017.pdf]

Reviewer #1:

The paper by Corman et al explores the effects of drugs on protein synthesis. The authors generated a high through put chemical screen to identify possible regulators of protein synthesis. Up regulators and down regulators. Based on this work two major discoveries were made: 1. Drugs do not promote protein synthesis above the level found under regular tissue culture conditions. 2. In addition to mTOR inhibitors, sphingosine kinase inhibitors downregulate protein synthesis in a mechanism that involves the induction of ER stress and eIF2a phosphorylation. This can be reversed by PERK inhibitors and the integrated stress response inhibitor ISRIB.

While this broad pharmacological analysis may be of general interest, I find this publication suitable better to a niche journal in pharmacology, such as JPET. This conclusion is based on the following general comments:

In the current version, we have added extra data that strengthen the manuscript and we now can provide: (a) the first available resource that documents how medically approved compounds affect global mRNA translation in living human cells. All previous chemical screens on translation were done *in vitro* and analyzing the effects on a single reporter. (b) We identify a new class of compounds that lower translation in human cells, and (c) we unambiguously demonstrate that the cell killing properties of SPHK inhibitors are surprisingly independent of SPHKs. I think this last point is particularly relevant as these agents are being used today in human clinical trials. With all of the above, I hope this reviewer shares that it merits publication in PLoS Biology.

1. The initial screen identified 54 drugs that potentially induce protein synthesis in U2OS cells. A second validation excluded all. This put a big question mark on the validity of the screen, as the authors acknowledge. The authors should look for conditions that avoid these false positive outputs.

We now discuss on this extensively on the manuscript. The setup that we used (human cancer cells grown in complete media with 10%FCS) made it very challenging to identify compounds that can upregulate translation in these conditions. We don´t even see an effect of insulin, which is widely accepted to upregulate translation.

Consistently, the primary hits for the screen on upregulating translation were selected based on effects much more modest than those observed for compounds lowering it. I would argue that the fact that mTOR inhibitors were consistently found among the translation downregulators speaks positively about the robustness of our approach.

In addition to discussing on these topics, in the current version we included data that illustrates that the effects of insulin in upregulating translation are only observed in cells undergoing starvation (Fig. S1). In fact, we know of no condition, chemical or genetic, that has been ever reported to upregulate translation in cells where translation was not previously lowered by starvation or stress. It would definitely be interesting to conduct another screen in starved cells to address which compounds can rescue translation in this condition but acknowledging that does not diminish the validity of our work.

2. The authors use a concentration of 10uM of the SK inhibitors. While this is not a tremendously high concentration, it is considerably high for kinase inhibitors. Dose responses and relevance to the clinical concentrations should be done. These data should be accompanied by analysis of sphingosine phosphorylation to determine if this is an off target effect of the drug. Many drugs induce ER stress at supra-pharmacological concentrations (celecoxib for example), a feature that is not related to the drugs primary pharmacological activity. This needs to be validated. Indications that sphingosine kinase inhibitors are inducing ER stress were already documented, so this is not a novel finding.

First of all, we actually show that compounds lower translation in a dose-dependent manner starting at 1 μM. While we used 10μM in our subsequent studies, this is the standard dose that has been used with these inhibitors in previous publications related to its role as a SPHK inhibitor. As to the novelty, to our knowledge no publications have been reported on the capacity of SPHKs to lower translation. In any case, in the current version of the manuscript we provide formal proof that the effects of SK-II and its clinical derivative ABC294640 on translation but also -importantly- on cancer cell killing are equally observed in cells lacking SPHK1 and SPHK2. I hope the reviewer shares the relevance of these findings for the development of these agents as medical drugs.

3. The fact that no drugs induced protein synthesis is important. However, this must be put in a more physiological context. For example, DMEM contains ample of amino acids, while serum does not. It is plausible that under ample of AA, glucose and serum supplementation the cells maximize protein synthesis. The authors should think about a relevant condition in which drugs may downregulate this and then repeat the screen under more stringent conditions. This may increase the impact of the study.

This relates to our response to the 1st comment. We actually agree that completing another screen in more stringent conditions (e.g. starvation) would certainly be an interesting follow-up for our work which we are already considering. However, I hope the reviewer agrees in that completing such screen, with its validations etc... is an entirely new project that would demand more work and time that what is available for a review process.

Minor issues:

The authors should quantify the expression and activity of SPHK 1 and 2 in their cells. This is important to conclude whether the two kinases are affecting similarly the stress responses.

This comment is responded by our novel data in SPHK1/2-deficient cell lines generated by CRISPR-Cas9.

The ATF4 antibody has background which may affect the immunofluorescence credibility. Please include a validation of the staining.

This antibody has been used in numerous publications and we believe that the

translocation data is rather clear. Moreover, the fact that the translocation is reverted by PERKi or ISRIB provides further support that we are evaluating the activation of the ISR, which is the purpose of these data. Finally, we not only provide images, as our ATF4 imaging data is entirely concordant with the WB data which we also provide for ATF4.

The fact that newly synthesized proteins are found in the nucleus is not surprising after 3 or 24 hours of labeling. Why the nuclear proteins were excluded from the analysis? Do the authors see cytoplasmic proteins only under shorter pulse conditions? Why the conditions of the screen were determined as done? This deserves explanation.

First of all, the OPP pulse was done for 30 min, not 3 or 24 hrs. As now clearly indicated in the manuscript, we focused on the cytoplasmic OPP signal as mRNA translation occurs primarily in the cytoplasm, and the nuclear OPP signals have been previously documented to be a result of abortive translation (Uozumi et al. 2016).

In any case, in addition to explaining this point, we now have provided the crude data that illustrates that using total or cytoplasmic OPP signals does not substantially differ in its capacity to identify compounds regulating translation (Fig. 1C).

The authors should quantify total ceramide levels in the cells when ER toxicity is observed. Is it due to impairment in ceramide levels (very low in the ER) or is it something else? Is it related to the signaling of S1P? This may add more mechanistic understanding to the phenomenon.

Again, this comment is responded by our novel work providing the data in SPHK1/2-deficient cell lines generated by CRISPR-Cas9 which. Given the clinical trials that are now being done with ABC294640 (including for COVID-19), we believe that clarifying that the mechanism of action of these compounds are due to its believed target clarifying for the field and likely one of the most important contributions from our work.

Reviewer #2:

Translation is essential to support cell survival and proliferation and defects in the control of this cellular process were linked to the development of several diseases. Translation requires significant amount of energy and this biological process is notoriously known to be regulated by the presence of growth factors and nutrients. Over the years, several groups have shown that the PI3K/mTOR pathway, a signaling node that senses nutrients and growth factors, plays a central role in regulating translation. Whether translation can be regulated by other pathways is still elusive. Here, Corman et al performed a chemical screen to identify new pharmacological regulators of translation. This screen, performed using more than 4000 compounds, confirmed that PI3K/mTOR inhibition strongly represses translation in vitro. None of the compound tested consistently increased translation. Here, the authors report that sphingosine kinase (SPHK) inhibitors significantly reduced translation in cells.

The authors show that SHPK inhibitors repress translation by altering ER membranes integrity and by activating the UPR. Overall, this manuscript is well written and the results presented are clear. The experimental approaches are well described and appropriate. The conclusions drawn by the authors are, most of the time, supported by the data presented. The major concern I have regarding this work relates to the novelty of the results presented. The most important regulators of translation identified from the screen are part of the PI3K/mTOR pathway, a signalling node that was already established as a key player in regulating translation. The other molecules that showed an effect on translation were inhibitors of SHPK, a set of molecules that were previously shown to activate the UPR. Considering the well-established roles of the UPR on the repression of translation, it is not clear to me how the current manuscript provides real conceptual advances inthe understanding of the cellular processes that control translation. Defining the precise impact of SPHK inhibitors on the lipid composition of the ER membranes and trying to determine how these changes translates into the activation of the UPR could represent a great addition to this paper.

We thank the reviewer for his/her nice comments on our word and for stating that our MS is well written, the results clear and the conclusions supported by the data. As for the novelty, we would want to note that:

(1) This is the first available resource that documents how medically approved compounds affect global mRNA translation in living human cells. All previous chemical screens on translation were done *in vitro* and analyzing the effects on a single reporter.

(b) Despite the link of SPHK inhibitors to the UPR, no report has documented that these compounds downregulate translation.

(c) Importantly, in the current version we unambiguously demonstrate that the ISR-activation and cell killing properties of SPHK inhibitors are surprisingly independent of SPHKs. I think this last point is particularly relevant as these agents are being used today in human clinical trials.

With all of the above, I hope this reviewer shares that it merits publication in PLoS Biology.

1) The authors mention that a part of the OPP signal is located to the nucleus and then conclude that 'as translation takes place in the cytoplasm, we used the quantification of cytoplasmic OPP signal for subsequent analysis'. Because OPP labelled proteins can theoretically diffuse in the cell to reach other cellular compartments, it is not clear to me why only the cytosolic amount of OPP signal was quantified. More explanations should be provided.

As now clearly indicated in the manuscript, we focused on the cytoplasmic OPP signal as mRNA translation occurs primarily in the cytoplasm, and the nuclear OPP signals have been previously documented to be a result of abortive translation (Uozumi et al. 2016).

In any case, in addition to explaining this point, we now have provided the crude data that illustrates that using total or cytoplasmic OPP signals does not substantially differ in its capacity to identify compounds regulating translation (Fig. 1C).

2) Have the authors measured the linearity of the OPP staining before running the screen? This should be discussed.

Yes, we have, and OPP (or HPG-labelling) provide linear measurements of translation as can be seen by the dose-dependent responses of controls such as mTOR inhibitors.

3) It is not clear to me how 54 compounds, identified as up-regulators of translation in the primary screen, all failed in the replication screen. This indicates that either i) the criteria to include these molecules as hits in the primary screen were too inclusive or ii) that a problem occurred in one the screen performed (primary or validation). The authors should comment.

We now discuss on this matter extensively on the manuscript. The setup that we used (human cancer cells grown in complete media with 10%FCS) made it very challenging to identify compounds that can upregulate translation in these conditions. We don´t even see an effect of insulin, which is widely accepted to upregulate translation.

As a consequence, the primary hits selected from our screen as potentially upregulating translation were based on effects much more modest than those observed for compounds lowering it. I would argue, however, that the fact that mTOR inhibitors were consistently found among the translation downregulators speaks positively about the robustness of our approach.

In addition to discussing on these topics, in the current version we included data that illustrates that the effects of insulin in upregulating translation are only observed in cells undergoing starvation (Fig. S1). In fact, we know of no condition, chemical or genetic, that has been ever reported to upregulate translation in cells where translation was not previously lowered by starvation or stress.

4) The primary screen performed here to identify new regulators of translation was based on the use of 4,166 molecules. The most important regulators of translation identified from this screen were part of the PI3K/mTOR pathway, a signaling node that was already established as a key player in regulating translation. Overall these results were expected based on previous literature. The other molecules that showed an effect on translation were inhibitors of sphingosine kinase, a set of molecules that were previously shown to activate the UPR. Considering the well-established roles of the UPR on the repression of translation, it is not clear to me how the current manuscript provides real conceptual advances in the understanding of the cellular processes that control translation.

We agree that the manuscript did not identify any pathway that lowered translation below what is seen with mTOR inhibitors. That being said: (a) that finding, in itself, is interesting; (b) we provide the first systematic characterization of how medically approved compounds influence translation; (c) while it could be expected, the fact that SPHK inhibitors lower translation has actually not been shown.

5) Defining the precise impact of SPHK inhibitors on the lipid composition of the ER membranes and trying to determine how these changes translates into the activation of the UPR could represent a great addition to this paper.

We agree with the reviewer even though our primary purpose was to provide a general characterization of the effects that medicines on translation. In any case, our current version of the manuscripts includes data on SPHK1- and/or SPHK2-deficient cells which clearly shows that SK-II and its clinical derivative ABC294640 activate the ISR and kill cells independently of SPHKs. Given the clinical trials that are now being done with ABC294640 (including for COVID-19), we believe that clarifying that the mechanism of action of these compounds is not due to its accepted targets should be clarifying and is one of the most important contributions from our work.

6) Are the effects of Torin2 and SKI-II on translation additive? If SKI-II controls translation by activating the UPR, independently of mTOR, we could expect to see an additive effect of both compounds.

Yes, they are, and these data are now provided in the MS (Fig. S2A).

7) It would be interesting to probe for GRP78 levels in the experiment described in 2C and 2E.

Thanks for the suggestion. We believe that anyhow the activation of the UPR by SK-II is nevertheless already strongly supported by our proteomic data (Fig. 3), which indicate that this is a likely the main cellular effect of SK-II.

8) The phospho-PERK blot presented in Figure 2E is difficult to interpret. Another WB with an anti-phospho PERK antibody should be presented. Several antibodies against pPERK are commercially available.

We have now provided a new blot to evaluate PERK phosphorylation (Fig. 2E).

9) After a careful review of the manuscript, I really think the authors should revise the title of their manuscript to better describe their findings and to be more specific. Here, the authors used a chemical screen and found that sphingosine kinase inhibition promotes UPR activation and represses translation. The current title of the manuscript 'A chemical screen identifies a link between metabolism and mRNA translation' is somehow misleading and does not summarize the data presented.

Based on our new data with SPHK-deficient cells we have now changed the title which now reads: "A chemical screen for modulators of mRNA translation identifies a novel mechanism of toxicity for sphingosine kinase inhibitors". We think this title better represents two of the main messages from our work: (a) the first chemical screen on overall translation done in living human cells and (b) the fact that SPHK inhibitors kill cancer cells regardless of SPHK1 and SPHK2.

10) In the abstract and in the discussion, the authors mention 'On the other hand, and despite the large number of molecules tested, our study failed to identify chemicals substantially increasing translation, raising doubts on to what extent translation can be supra-physiologically stimulated in mammalian cells'. I do not think this conclusion is correct. The incapacity to promote translation to a very high level is likely specific to the experimental model used. Here, all the experiments were performed in proliferating cancer cells grown in rich conditions (high energy, full serum). These cells likely maximize basal translation to support proliferation. Because the authors did not measure translation in normal mammalian tissues and in response to conditions that may impact on translation (ex. cell differentiation, cell proliferation, cell activation), it is incorrect to say that translation cannot be highly induced in mammalian cells.

This comment relates to the third comment and is now discuss on this extensively on the manuscript. The reviewer is absolutely right in that the setup that we used (human cancer cells grown in complete media with 10%FCS) made it very challenging to identify compounds that can upregulate translation in these conditions. We don´t even see an effect of insulin, which is widely accepted to upregulate translation. In addition to discussing on these topics, in the current version we included data that illustrates that the effects of insulin in upregulating translation are only observed in cells undergoing starvation (Fig. S1). In fact, we know of no condition, chemical or genetic, that has been ever reported to upregulate translation in cells where translation was not previously lowered by starvation or stress.

It would definitely be interesting to conduct another screen in starved cells to address which compounds can rescue translation in this condition but acknowledging that does not diminish the validity of our work.

Minor
1) The authors should produce a table clearly listing the 48 compounds that decreased and the 54 compounds that increased translation.

The hits were already annotated in our original Table S2. In any case, as requested, we now also provide these data in a dedicated table, although we want to note that the hits from a primary screen should always be taken with caution before subsequent validation data are obtained.

Reviewer #3:

In the current manuscript, Corman et al. developed a chemical screening approach to identify small molecule modulators of general translation in a human cancer cell line (U2-OS). The authors tested a library of more than 4,000 compounds, but could not identify translational activators. However, they were able to recovered inhibitors of the mTOR/PI3K pathway as potent translational repressors. In addition, the authors identified sphingosine kinases, especially SPHK2, as molecular targets to block translation. The latter effect seems to depend on the induction of ER stress and the integrated stress response by the sphingosine kinase inhibitors. Consequently, the effects could be reversed by the simultaneous inhibition of both sphingosine kinase and PERK.

Overall, the manuscript is well-written and easy to follow and the results are of general interest to the RNA community. Nevertheless, I have some concerns that the authors should try to address:

We thank the reviewer for his/her nice words on our work. In the current version we have added new data, including the suggestion of using SPHK-deficient cell lines made by CRISPR that have turned out to be one of the most important messages from our work.

1) Please elaborate a bit more on the selection and composition of the chemical library. Why were these inhibitors chosen? How many kinase inhibitors vs. epigenetic inhibitors vs. others, etc.?

Information on the chemical library was already provided in the Materials and Methods Section. We mainly wanted to characterize the effects of medically approved drugs on translation, for which we used the Prestwich Chemical Library of FDA approved compounds (circa 1,200). While this was our primary objective, we decided to add other ad-hoc libraries which included tool compounds for which the target is known (Selleck tool, ENZO tool or Tocris mini), a library with all available kinase inhibitors (Selleck known kinase inhibitors), a library of Bromodomain inhibitors given the effects observed for some of them in targeting MYC (e.g. BRD4 inhibitors) and 115 covalent drugs synthesized in house in search of potential new compounds that could regulate translation through unexpected ways.

2) Please improve the workflow scheme in Figure 1C. The pulse labelling with OPP for 1 h is not obvious and also the second incubation phase is 24 h, after 23 h CHX was added. Is this information really needed here? Also, it has to be explained why the nuclear counterstaining was done. It is just briefly indicated with (viability) in the text. Last but not least, the choice of the cell line system should be explained. I guess U2-OS were selected due to their rather large cytoplasmic compartment, correct? However, please see my next point.

U2OS was chosen since it is a widely used cancer cell line and as noted by the reviewer its cytoplasm/nuclear ratio and its adherent properties make it very useful for image-based High Throughput screens. As for Figure 1C, we have modified it to make it more clear for the readers as to how the screen was performed. The nuclear co-

stain was used both to evaluate the effects of the compounds on viability but also to enable the quantification of the cytoplasmic OPP signal.

3) Only the cytoplasmic OPP signal was considered for the screening analysis. However, proteins move to their target destination after synthesis. Hence, the whole-cell signal should be considered. How would this differ from the current analysis?

As now clearly indicated in the manuscript, we focused on the cytoplasmic OPP signal as mRNA translation occurs primarily in the cytoplasm, and the nuclear OPP signals have been previously documented to be a result of abortive translation (Uozumi et al. 2016).

In any case, in addition to explaining this point, we now have provided the crude data that illustrates that using total OPP signals or cytoplasmic does not substantially differ in its capacity to identify compounds regulating translation (Fig. 1C).

4) Please improve some of the Western blot images to allow more band width. For example, in Figure 2E the signals for PERK are nearly at the border / edge of the box. Also, a black line is visible. In line with this, Figure S1C should also show a larger section of the blot.

We have provided new western blots and eliminated the lines from WB panels.

5) The damage of SKI-II to the ER membranes happens quite fast (after 3-6 h). However, it is unclear what really happens to them. The authors did not measure any lipids. Do these change?

See our next response, the effects of SKI-II or ABC294640 on ER membranes are unrelated to SPHKs.

6) How specific is the SKI-II or ABC294640 inhibitor? Is the effect truly mediated by SPHK2 inhibition? How effective is the inhibition, i.e. can the authors measure remaining activity? To analyze specificity one usually leverages genetic (knockout) models. The inhibitor should not cause any effects in such a system. Also, the authors should perform SPHK2 depletion experiments and analyze the effects on translation and stress pathways.

This was an excellent suggestion which has become what I believe is one of the most important messages from our work. Following the reviewers´ advice we have now generated SPHK1- and/or SPHK2-deficient cells. Surprisingly, our data shows that SK-II and its clinical derivative ABC294640 activate the ISR and kill cells independently of SPHKs. Given the clinical trials that are now being done with ABC294640 (including for COVID-19), we believe that clarifying that its mechanism of action is not due to SPHKs is in fact one of the most important contributions from our work.

7) Please discuss the obvious lack of ABC294640 activity in your screen and its clinical relevance given that effects were only achieved with a much higher (50 μM) dose.

The doses we use for ABC294640 are in the range of those used in other publications using this drug and the ones required to kill cancer cells. As for why this compound did not come as a hit in our screen, this is simply because it was not part of the library as it is not yet medically approved.
