# Supplementary material for: A chemical screen for modulators of mRNA translation identifies a distinct mechanism of toxicity for sphingosine kinase inhibitors
Source: PLoS Biol. 2021 May 25;19(5):e3001263. doi: 10.1371/journal.pbio.3001263 (PMC8183993; doi:10.1371/journal.pbio.3001263)
Supplement: Attachment — Submitted filename: Point_by_point[OPP].pdf [file pbio.3001263.s018.pdf]

Reviewer #1:

I don't have any further scientific comments. The fact that drugs have off target effects at high concentrations is not unusual. The authors indicate the development of a chronic ER stress condition that merits caution.

I followed the clinical trial data and it seems that ABC294640 at 500 mg per day is tolerated well. If this molecule would cause chronic ER stress conditions, I assume that blood glucose levels would have risen. No reports were documented. I think that the lack of a mechanism by which SKII inhibitors is working off-target detracts my enthusiasm. My opinion is that PLOS Biology is seems to be above the scientific significance of these findings.

The reviewer here focuses his/her only criticism in that he/she does not believe that reporting that the toxicities of two sphingosine kinase inhibitors (SKI-II and ABC294640) are related to the activation of the ISR and to effects of translation by these compounds, independently of SPHK1 and SPHK2. He/she bases the criticism in that this might be related to high doses of the compound used, and if that if our findings were to be clinically relevant one would expect to see certain toxicities in patients.

Our response to this comment follows:

#1 First, the reviewer ignores the rest of our manuscript, where we provide the first resource that illustrates how every medically approved compound affects overall translation levels in mammals.

#2 Second, and as mentioned in our previous response, the doses we used in our work are equivalent to those that have been used in previous manuscripts evaluating the toxicity of sphingosine kinase inhibitors. Note, for instance, that we already detect an effect of SKI-II in translation at 1 μM.

#3 Finally, I think it is far stretched to make conclusions about the mechanism of action of a drug based on clinical observations. Topoisomerases are essential enzymes, yet etoposide or irinotecan have yielded nearly curative responses in multitude of cancer patients. Our work indicates that SPHK inhibitors, including the clinical ABC294640, can kill cancer cells independently of its sphingosine kinases. We believe that the scientific community, particularly those most actively involved in the clinical development of this therapy, should be aware of it. We are sorry if the reviewer believes that this it not scientifically significant, we disagree.

Reviewer #2:

I want to thank the authors for this new version of the manuscript. They provided answers for most of my comments. Here are minor comments that should be addressed.

We thank the reviewer for his/her nice words on our work.

Comments on the revised manuscript.

1) The authors should review the abstract to remove this sentence: 'None of the compounds upregulated translation, which could be due to the screen being performed in cancer cells grown in full media where translation is already present at very high levels'. This information is not useful in the abstract.

While we understand the reviewers´ point of view, we still believe that at least some of the readers might find it interesting that for cancer cells grown in full media, none of the known medicines seem to upregulate translation.

2) Line 6 - change mammalian for mechanistic.

Done, thanks for spotting it!

3) Loading control S4C is weird (and the levels of SPHK2 are highly variable). The authors should provide explanation or run the experiment again.

The loading control for Figure S4C is actually vinculin, not SPHK2. For the analysis of SPHK2 levels we used in-cell western blotting, which we find is not always trivial to optimize for the equal loading. In any case, the control for SPHK2 levels is GAPDH (also done by in-cell WB), and the only purpose of this part of the pannel is to illustrate that SPHK2-KO and SPHK1/2-KO cells are in fact deficient for SPHK2 expression which is already shown in **Fig. 4C.** In any case, we have now repeated the WB, and provided an updated **S4C** which is much clearer.

4) Uniformize the use of SK-II and SKI-II thought the manuscript.

Thanks for this suggestion. Indeed, the actual name of the compound is SKI-II and we have now used it throughout the MS.

5) Figure 2C - why does Torin 2 reduces total S6K levels? Are the authors sure that the right antibody was used? Looks like pS6K is presented instead of total S6K.

We in fact mistakenly used the pSK6 blot twice in this panel, one of which has been now removed. Total S6K levels were simply used as loading controls, and this panel also shows the levels of Vinculin and eIF2a, which are equally valid for this purpose. We thank the reviewer for noticing this.

Comment on the responses provided.

Comment 9 : The title suggested is better but still not accurate. This title 'A chemical screen for modulators of mRNA translation identifies a novel mechanism of toxicity for sphingosine kinase inhibitors' is incorrect considering that the mechanism of toxicity is not identified/provided. The data presented indicate that SPHK inhibitors blocks translation independently of SPHK1/2, but the mechanism is still unclear.

While we do not know the target that mediates the sphingosine-kinase independent toxic effects of SKI-II and ABC294640, we believe that this not invalidate the fact that our work reveals the mechanism of toxicity for these compounds is different to the previously accepted one, and thus novel.

Reviewer #3:

The authors have fully answered my previous concerns and I have no further questions. However, they need to include the sgRNA sequences used to generate SPHK1/2 CRISPR KOs.

We thank the reviewer for his/her assessment. The sgRNA sequences targeting SPHK1 and SPHK2 are now provided in the manuscript.
